# How prevalent are modifiable dementia risk factors in Ireland? A 12-year observational study in community-dwelling older adults

**DOI:** 10.1136/bmjopen-2025-106032

**Published:** 2025-11-11

**Authors:** Caoimhe McGarvey, Rose Anne Kenny, Sean Kennelly, Donal Sexton, Robert Briggs

**Affiliations:** 1 The Irish Longitudinal Study on Ageing, Trinity College Dublin, Dublin, Ireland; 2 Mercer’s Institute for Successful Ageing (MISA), St James’s Hospital, Dublin, Ireland; 3 Department of Medical Gerontology, Trinity College Dublin, Dublin, Ireland; 4 Trinity Centre for Health Sciences, St. James’ Hospital, Dublin, Ireland; 5 Tallaght University Hospital, Dublin, Ireland; 6 Dementia Research Network Ireland (DRNI), Dublin, Ireland; 7 School of Medicine, Trinity College Dublin, Dublin, Ireland; 8 Renal Unit, St James’s Hospital, Dublin, Ireland

**Keywords:** Dementia, Risk Factors, Cognitive dysfunction, Cardiovascular Disease

## Abstract

**Abstract:**

**Objectives:**

Dementia is potentially preventable and deferrable yet remains a major cause of disability, dependency and mortality worldwide. The 2024 Lancet Commission on dementia identified 14 modifiable dementia risk factors and estimated that addressing these could reduce dementia cases by up to 45%. The aim of this study is to assess dementia risk factor prevalence in adults ≥50 years participating in a nationally representative longitudinal study on ageing, providing crucial context for the delivery of dementia prevention.

**Design and setting:**

The Irish Longitudinal Study on Ageing (TILDA) is a population-based prospective cohort study, representative of community-dwelling adults ≥50 years living in Ireland.

**Participants:**

All participants from waves 1 (2009–2010): n=8171, 3 (2014–2015): n=6615 and 6 (2021–2023): n=4318 of TILDA were analysed over a 10.93 (±0.37) years of follow-up.

**Results:**

70.6%, 61.1% and 54.2% of the population had ≥4 modifiable risk factors for dementia at consecutive waves, amounting to over 500 000 people with ≥4 modifiable risk factors for dementia on weighted population analysis at wave 6. 77% of those with severe decline in cognitive performance during follow-up had ≥4 risk factors at baseline. An estimated 32 480 cases of severe decline in cognitive performance during follow-up were potentially preventable if risk factors were addressed.

**Conclusions:**

In a nationally representative sample of older European adults, there is a high prevalence of modifiable dementia risk factors. This highlights the need for greater attention on educating people on the concept of brain health through public health messaging as well as the development of a clinical framework focused on delivering on the opportunity of dementia prevention. Preventing and delaying dementia onset can have a significant impact on the compression of morbidity and increasing healthy lifespan in older age.

**STRENGTHS AND LIMITATIONS OF THIS STUDY**

- The strengths of this study include the involvement of a large, population-representative cohort of community-dwelling older adults.
- Comprehensive data collection, standardised health assessments and ongoing follow-up with extensive longitudinal data further enhance the robustness of this study.
- This study has some limitations; some of the variables, including established cardiovascular disease and diabetes status, are based on participants’ self-report of a doctor-delivered diagnosis, which could be subject to recall bias.
- In terms of hypertension measurement, it is well documented that blood pressure readings can be inappropriately high or low in healthcare settings, termed either ‘white coat hypertension’ or ‘masked hypertension’; this may cause overestimation or underestimation of hypertension prevalence.
- Additionally, cognitive decline often causes participants to withdraw from longitudinal studies and this may lead to an underestimation of dementia risk.

## Introduction

 Dementia is an umbrella term for a group of neurodegenerative diseases, primarily affecting memory and cognitive function, that significantly impact a person’s ability to carry out their activities of daily living.^1^ Dementia is a major cause of disability and dependency among older adults worldwide and is now the seventh leading cause of death.^2^ The number of people living with dementia in 2019 was estimated to be 57 million and is projected to increase to 153 million globally by 2050.^3^ Evidence suggests that dementia can be both preventable and deferrable as age-specific dementia incidence rates are falling in high-income countries.^4^ Preventing and delaying dementia onset has the potential to have a significant impact in terms of the compression of morbidity and increasing healthy lifespan in older age.^4^

In July 2024, the Lancet Commission on dementia published an updated report on dementia prevention, intervention and care. This report identified 14 modifiable risk factors for dementia based on the most up-to-date evidence and estimated that there could be a 45% reduction in cases of dementia if these risk factors were addressed.^5^ The risk factors identified were lower educational attainment, hearing loss, high low-density lipoprotein (LDL) cholesterol, hypertension, physical inactivity, diabetes, social isolation, excessive alcohol consumption, air pollution, smoking, obesity, traumatic brain injury, depression and visual loss. The aim of this study is to assess the prevalence of these modifiable risk factors in the older, community-dwelling population in Ireland with a view to informing national policy and practice in terms of dementia prevention in Ireland.

## Methods

### Study setting and participants

This study is longitudinal in design using data from the first, third and sixth waves of the Irish Longitudinal Study on Ageing (TILDA). TILDA is a population-based prospective cohort study, representative of community-dwelling adults aged 50 years and older, living in Ireland. The sample was recruited in geographic clusters, based on a national directory of residential addresses in Ireland, using the RANSAM sampling system.^6^ Each member of the population in Ireland aged 50 years and older and living in the community had an equal probability of being invited to participate.

Participants were invited to complete a computer-assisted personal interview (CAPI), a Self-Complete Postal Questionnaire (SCQ) and a health centre or home-based health assessment (HA).^7^ Data collection in wave 1 was carried out from 2009 to 2011 and included 8501 participants, wave 3 data collection took place from 2014 to 2015 and included 6687 participants and data collection in wave 6 took place from 2021 to 2023 and included 4332 participants. The mean (±SD) follow-up period was 10.93 (±0.37) years, ranging from 10.02 to 12.86 years.

### Risk factor measurements

As summarised in table 1, the highest education level achieved was recorded in the CAPI, less educational attainment was defined as primary education or no education. Self-rated hearing was recorded in the CAPI, hearing loss was defined as a rating of fair or poor hearing or as self-reporting hearing loss and/or the use of hearing aids. Self-rated hearing was previously validated in the TILDA study and its reliability compared with the Whispered Voice Test was confirmed.^8^ Self-rated vision and usual use of glasses or contact lenses were recorded at the CAPI and visual loss was defined as a rating of poor or fair. Diabetes status was recorded in the CAPI and was defined as a self-reported doctor-delivered diagnosis of diabetes. Problematic alcohol consumption was defined as a score of >2 on the Cutdown, Annoyed, Guilt, Eye-opener questionnaire or as self-reported alcohol abuse.^9^ Smoking status was recorded as never, past or current smoker at the CAPI and both current and past smoking were defined as a risk factor for dementia.

**Table 1:** Risk factor measurement

Risk factor	Data source	Data measure in TILDA	Risk factor identified	Self-report or objective	Data measure in studies in the Lancet Commission Report
Lower educational attainment	CAPI	Highest educational level achieved	Primary education or no education	Self-report	Educational attainment
Hearing loss	CAPI	Self-rated hearing or hearing loss Use of hearing aids	Poor or fair Yes	Self-report	Objective measure with pure-tone assessment
Visual loss	CAPI	Self-rated vision Self-report of visual problem Frequent use of glasses or contact lenses	Poor or fair Yes Yes	Self-report	Visual acuity, self-reported
Diabetes	CAPI	Self-reported doctor-delivered diagnosis	Yes	Self-report	Clinical diagnosis
Excessive alcohol consumption	CAPI	CAGE questionnaire	CAGE>2	Self-report	Weekly alcohol intake in units
Smoking	CAPI	Self-reported smoking history	Current or smoking history	Self-report	Current or smoking history
Physical inactivity	CAPI	IPAQ short form	Low physical activity	Self-report	Self-report and questionnaires
Social isolation	CAPI	Berkman-Syme Social Network Index	Score 0–1 (‘most isolated’)	Self-report	Any measure of social engagement or loneliness
Depression	CAPI	CES-D-20 CES-D-8 Self-reported doctor-delivered diagnosis Use of antidepressant medication	Score≥16 Score≥9 Yes Yes	Self-report Objective	Clinical diagnosis of depression or symptoms of depression measured by CES-D
Air pollution	SCQ	Home heating method	Open fire use	Self-report	Particulate matter concentration
High LDL cholesterol	HA	Blood sample	>3 mmol/L	Objective	High LDL cholesterol (ie, >3 mmol/L)
Obesity	HA	Body mass index Waist/hip ratio Waist circumference	>30 kg/m^2^ Male: >0.9 female: >0.85 Male: >102 cm female: >88 cm	Objective	BMI, waist circumference and waist-to-hip ratio
Hypertension	HA	Digital oscillometric measurement Use of antihypertensive medication	Mean systolic BP≥140 or mean diastolic BP≥90 Yes	Objective	Elevated systolic BP≥140
Traumatic brain injury	N/A	Not available	Not available	Not available	N/A

BMI, body mass index; BP, blood pressure; CAGE, Cutdown, Annoyed, Guilt, Eye-opener; CAPI, computer-assisted personal interview; CES-D, Centre for Epidemiological Studies Depression Scale; HA, health assessment; IPAQ, International Physical Activity Questionnaire; LDL, low-density lipoprotein; SCQ, Self-Complete Postal Questionnaire; TILDA, The Irish Longitudinal Study on Ageing.

Physical activity was self-assessed using the International Physical Activity Questionnaire (IPAQ) short form.^10^ The IPAQ scoring system categorises physical activity as low, moderate or vigorous/high intensity. Physical inactivity was therefore defined as low physical activity on the IPAQ for the purposes of this study. Social isolation was assessed using the Berkman-Syme Social Network Index.^11^ Scores using this index range from 0 to 4 (0–1=‘most isolated’ to 4=‘most integrated’), social isolation in this study was defined as a score of 0–1.

Depression was assessed in the CAPI using the Centre for Epidemiological Studies Depression Scale (CES-D).^12^ Either the original CES-D 20-item screening test with response values on 4-point Likert scales or the shortened CES-D 8-item scale was used. For the CES-D-20, a score of 16 or more is used to define depression compared with a score of nine or more for the CES-D-8.^13^ This shorter version of the CES-D scale was used in the TILDA study to reduce the time taken to conduct participant assessments, previous work has confirmed its reliability in comparison to the 20-item CES-D within the TILDA cohort.^14^ In this study, depression was defined as a CES-D-20 score of 16 or more, a CES-D-8 score of nine or more, a self-reported diagnosis of depression and/or taking antidepressant medications. Air pollution was recorded as a risk factor in those that self-reported using an open fire or a combination of an open fire and a portable heater to heat their home during winter in the SCQ. This question was not asked at wave 1, so the closest possible substitute, which was collected at wave 2 (2012 data collection), was used for wave 1 analysis. Other sources of air pollution were not assessed due to lack of availability in the dataset, the participants’ addresses are not routinely recorded as part of data collection and although geocodes are recorded, it was not possible to link them with an air pollution index.

In terms of measures collected at HA, LDL cholesterol was measured from each blood sample collected before freezing with an automated enzymatic method and a high LDL cholesterol was defined as LDL>3 mmol/L. Blood samples were available for 5630 participants at wave 1, 4767 participants at wave 3 and 2652 participants at wave 6. Height and weight were measured by the study health practitioner and body mass index (BMI) was calculated as weight in kg/height in m² and was available in 5869 wave 1 participants, 5294 wave 3 participants and 4321 participants. Obesity was defined as a BMI>30 kg/m², a waist/hip ratio>0.9 for males and >0.85 for females or a waist circumference>102 cm for males and >88 cm for females, depending on the measurement used at each wave of data collection.^15^

Blood pressure (BP) was measured by a healthcare practitioner as part of the HA, either in a health centre or in the participant’s own home. Measurements were taken according to a standard protocol at an ambient temperature of 20°C–25°C. A digital automated oscillometric BP monitor (Omron M10-IT, Omron, Kyoto, Japan) with an arm cuff (22–42 cm) was used to measure BP in one arm, at heart height, while the respondent was seated comfortably in an upright position after a period of rest. BP was recorded two times while seated with a timed interval of 1 min between readings. The mean systolic and diastolic readings were obtained from these two measurements.^16^ HTN was defined as a risk factor in those with a mean systolic BP≥140 or a mean diastolic BP≥90 and/or those taking antihypertensive medications. BP measurements were included for 5853 wave 1 participants, 5322 wave 3 participants and 3197 wave 6 participants. Traumatic brain injury was not assessed in this study.

### Covariates

Further demographic covariates included age and sex. Established cardiovascular disease (CVD) was also documented and was based on the self-report of a doctor’s diagnosis of one of the following conditions: angina, myocardial infarction, stroke, transient ischaemic attack, atrial fibrillation and heart murmur. The presence of multimorbidity and polypharmacy was recorded at each wave. Multimorbidity was defined as the presence of two or more chronic diseases, while polypharmacy was defined as taking five or more regular medications. Chronic diseases recorded in the dataset were diabetes, arthritis, cancer, liver disease, lung disease and heart failure. Mini-Mental State Examination (MMSE) and Montreal Cognitive Assessment (MOCA) scores were also recorded. Normative values for MMSE and MoCA in the TILDA study have been published previously.^17^

Impairments in both basic and instrumental activities of daily living were recorded in the CAPI. Interventions for hearing and vision loss, depression, diabetes and high LDL cholesterol were assessed. Participants reported in the CAPI if they wore hearing aids all the time or occasionally and if they usually wore glasses or contact lenses. The self-report of a doctor-delivered diagnosis of cataracts, glaucoma or age-related macular degeneration was also recorded. Antihypertensive, antidepressant, lipid-lowering and diabetic medication use were recorded during the CAPI and were classified according to the WHO Anatomical Therapeutic Chemical (ATC) classification system. Antihypertensive medication was defined as medications categorised as C02, C03, C07, C08 and C09, antidepressant medication was classified as N06A, lipid-lowering medication was classified as C10 and diabetic medication was categorised as A10 according to the ATC classification system. Participants who self-reported a mental health diagnosis were asked if they had received a psychological assessment in the CAPI.

### Decline in cognitive performance

To assess the potential impact of effective management and control of the modifiable risk factors for dementia, the decline in cognitive performance over the follow-up period was examined. Risk factor prevalence for this analysis was measured at wave 1 and cognition was measured at wave 1 baseline and at wave 6 follow-up. Three categories of change in cognitive performance: nil/mild, moderate and severe were created based on the change in MoCA score between waves one and six, over a mean follow-up period of 10.93 (±0.37) years. The first category (nil/mild) included those with less than one SD change in MoCA score, the second category (moderate) had a one to two SD change in MoCA score, and the third category (severe) included those with greater than two SD change in MoCA score. The group with a severe decline in cognitive performance was used to estimate the population-level impact of risk factor elimination over the follow-up period. Regarding dementia prevalence, estimates previously published by Pierse *et al* in 2019 were used.^18^

### Statistical analysis

Participant characteristics were presented as unweighted sample number (n), weighted percentage and 95% CI or mean and 95% CI. Descriptive statistics were used to calculate the prevalence of each risk factor individually and in combination. Inverse probability weighting was applied to account for the complex survey design used in TILDA. The population weights were calculated based on age, sex and educational attainment of the population in Ireland and aim to adjust for selection bias and non-response bias to the HA component of the survey. Further details on the weights applied are available elsewhere.^19^ Modifiable dementia risk factors were assessed across five categories; category 1 had no risk factors, category 2 had one to three risk factors, category 3 had four to six risk factors, category 4 had seven to nine risk factors and category 5 had ten or more risk factors. Data analysis was conducted using Stata V.15.1. Study investigators had secure access to the pseudonymised TILDA database which undergoes extensive validation checks as well as standardisation, anonymisation and quality assurance procedures.

### Patient and public involvement

TILDA has a patient and public involvement working group that contributed to the wave 6 data collection, but they were not directly involved in this study.

## Results

A total of 8171, 6615 and 4318 participants were included in the waves 1, 3 and 6 analyses, respectively. The mean (±SD) age of study participants at each wave was 63.8 (±9.7) at wave 1, 66.9 (±9.4) at wave 3 and 71.4 (±8.1) at wave 6. In wave 1, 54.2% of study participants were female, 55.6% were female at wave 3 and 56.9% were female at wave 6. The mean MoCA score of study participants across the three waves was 24.2 points. In terms of the dependency levels of the study cohort, 8.7% of study participants had at least one basic activity of daily living (ADL) impairment while 8.0% were identified as having at least one instrumental ADL impairment. Table 2 displays the weighted participant characteristics at each wave.

**Table 2:** Participant characteristics

Characteristic	Wave 1 N	Wave 1 weighted % and CI or mean	Wave 3 N	Wave 3 weighted % and CI or mean	Wave 6 N	Wave 6 weighted % and CI or mean
Age						
50–64	4664	58.1 (56.6 to 59.6)	3033	45.0 (43.4 to 46.6)	988	25.8 (24.0 to 27.7)
65–79	2879	32.0 (30.6 to 33.5)	2798	40.2 (38.7 to 41.7)	2594	57.2 (55.2 to 59.1)
80+	628	9.9 (9.0 to 10.8)	784	14.8 (13.6 to 16.1)	736	17.0 (15.6 to 18.4)
Sex						
Male	3743	48.2 (47.2 to 49.1)	2938	48 (46.9 to 49.1)	1860	47.8 (46.1 to 49.4)
Female	4428	51.8 (50.9 to 52.8)	3677	52.0 (50.9 to 53.1)	2458	52.2 (50.6 to 53.9)
Cardiovascular disease						
CVD history	4166	50.8 (49.3 to 52.4)	1524	23.1 (21.9 to 24.3)	1094	26.1 (24.5 to 27.8)
No CVD history	4005	49.2 (47.6 to 50.7)	5091	76.9 (75.7 to 78.1)	3224	73.9 (72.2 to 75.5)
Multimorbidity						
Multimorbidity	575	7.2 (6.6 to 7.8)	539	8.9 (8.1 to 9.7)	425	10.9 (9.8 to 12.2)
No multimorbidity	7596	92.8 (92.2 to 93.4)	6076	91.1 (90.3 to 91.9)	3893	89.1 (87.8 to 90.2)
Polypharmacy						
Polypharmacy	1682	21.2 (20.1 to 22.2)	1730	27.5 (26.2 to 28.8)	1617	38.9 (37.1 to 40.7)
No polypharmacy	6407	78.9 (77.8 to 79.9)	4885	72.5 (71.2 to 73.8)	2701	61.1 (59.3 to 62.9)
MMSE						
Mean	–	28.0 (27.9 to 28.1)	–	28.5 (28.4 to 28.6)	–	28.3 (28.1 to 28.4)
MoCA						
Mean	–	24.1 (24.0 to 24.3)	–	24.7 (24.5 to 24.9)	–	23.9 (23.6 to 24.1)
Evidence of cognitive impairment	3068	56.8 (55.2 to 58.3)	2431	47.8 (46.1 to 49.6)	948	59.5 (56.6 to 62.3)

CVD, cardiovascular disease; MMSE, Mini-Mental State Examination; MoCA, Montreal Cognitive Assessment.

**Figure 1:**
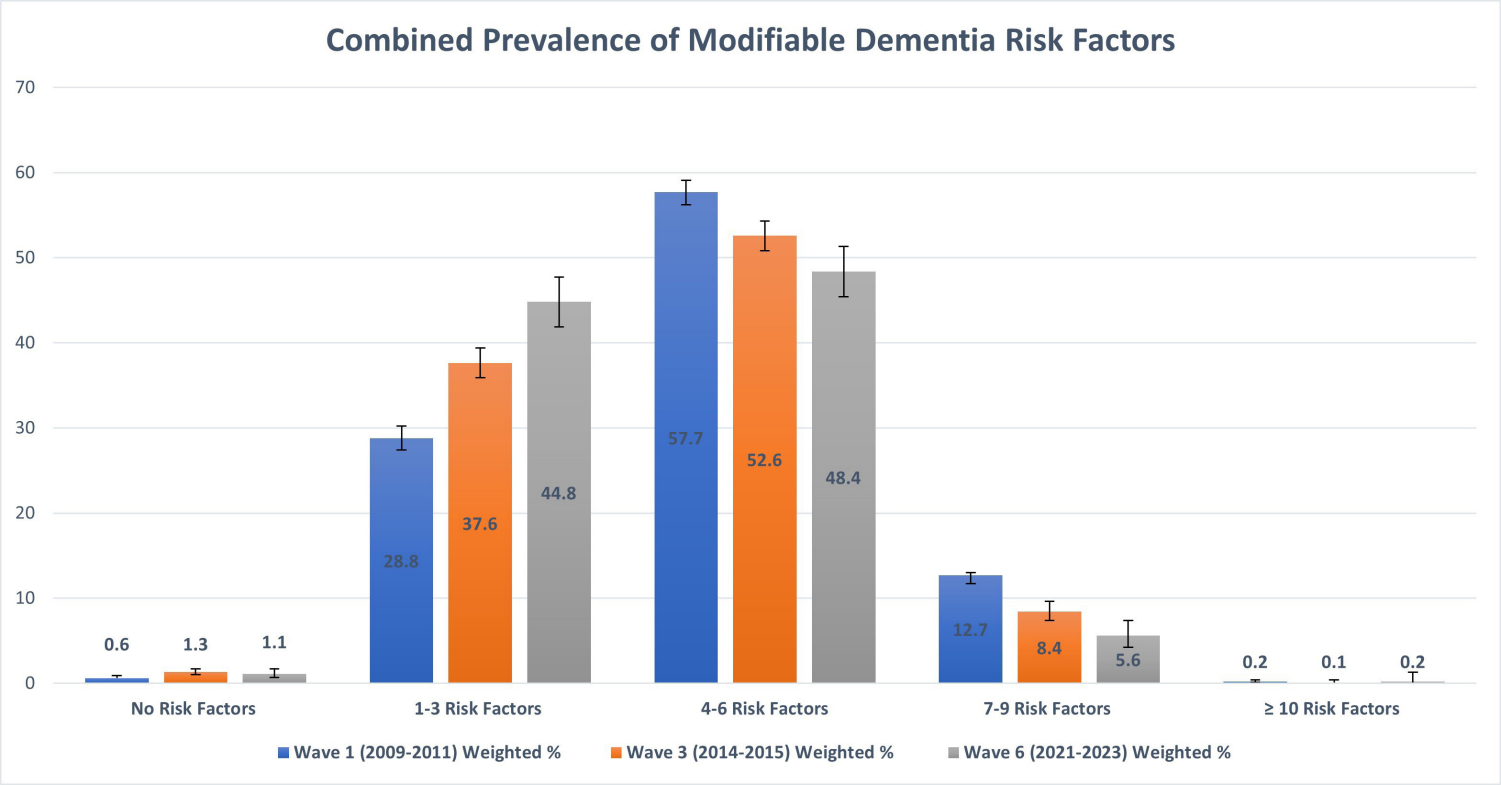
Combined prevalence of modifiable dementia risk factors in community-dwelling adults over 50 years in Ireland. This bar chart depicts the prevalence of modifiable dementia risk factors in community-dwelling adults over 50 years in Ireland at three time points: wave 1 (2009–2011), wave 3 (2014–2015) and wave 6 (2022–2023). The blue bars represent the data from wave 1, the orange bars represent the data from wave 3 and the grey bars represent data from wave 6. The number of risk factors is displayed on the x-axis and weighted prevalence for each group, expressed as a percentage, is displayed on the y-axis. Error bars represent the 95% CIs for each wave.

Regarding the prevalence of the modifiable risk factors for dementia identified by the Lancet Commission on dementia, 70.6% of the population had four or more risk factors for dementia at wave 1. This figure decreased to 61.1% at wave 3 and 54.2% at wave 6. On weighted analysis, over half a million people had at least four risk factors for dementia at wave 6. For context, based on 2022 census data, Ireland’s population of community-dwelling adults over 50 years old was estimated to be 1.1 million people. Table 3 and figure 1 provide a detailed breakdown of the prevalence of combined risk factors at each wave.

**Table 3:** Risk factor categories

Risk factor (RF) category	Wave 1 weighted % and CI	Population count	Wave 3 weighted % and CI	Population count	Wave 6 weighted % and CI	Population count
1 (no RF)	0.6 (0.5 to 0.9)	7862	1.3 (1.0 to 1.7)	15 244	1.1 (0.7 to 1.7)	11 420
2 (1–3 RF)	28.8 (27.4 to 30.2)	364 760	37.6 (35.9 to 39.4)	447 807	44.8 (41.9 to 47.7)	458 364
3 (4–6 RF)	57.7 (56.2 to 59.1)	731 563	52.6 (50.8 to 54.3)	625 731	48.4 (45.4 to 51.3)	495 483
4 (7–9 RF)	12.7 (11.7 to 13.8)	161 236	8.4 (7.4 to 9.6)	100 029	5.6 (4.2 to 7.4)	56 956
5 (≥10 RF)	0.2 (0.1 to 0.4)	2647	0.1 (0.0 to 0.4)	1183	0.2 (0.0 to 1.3)	1952
Total	100	1 268 068	100	1 189 993	100	1 024 175

**Figure 2:**
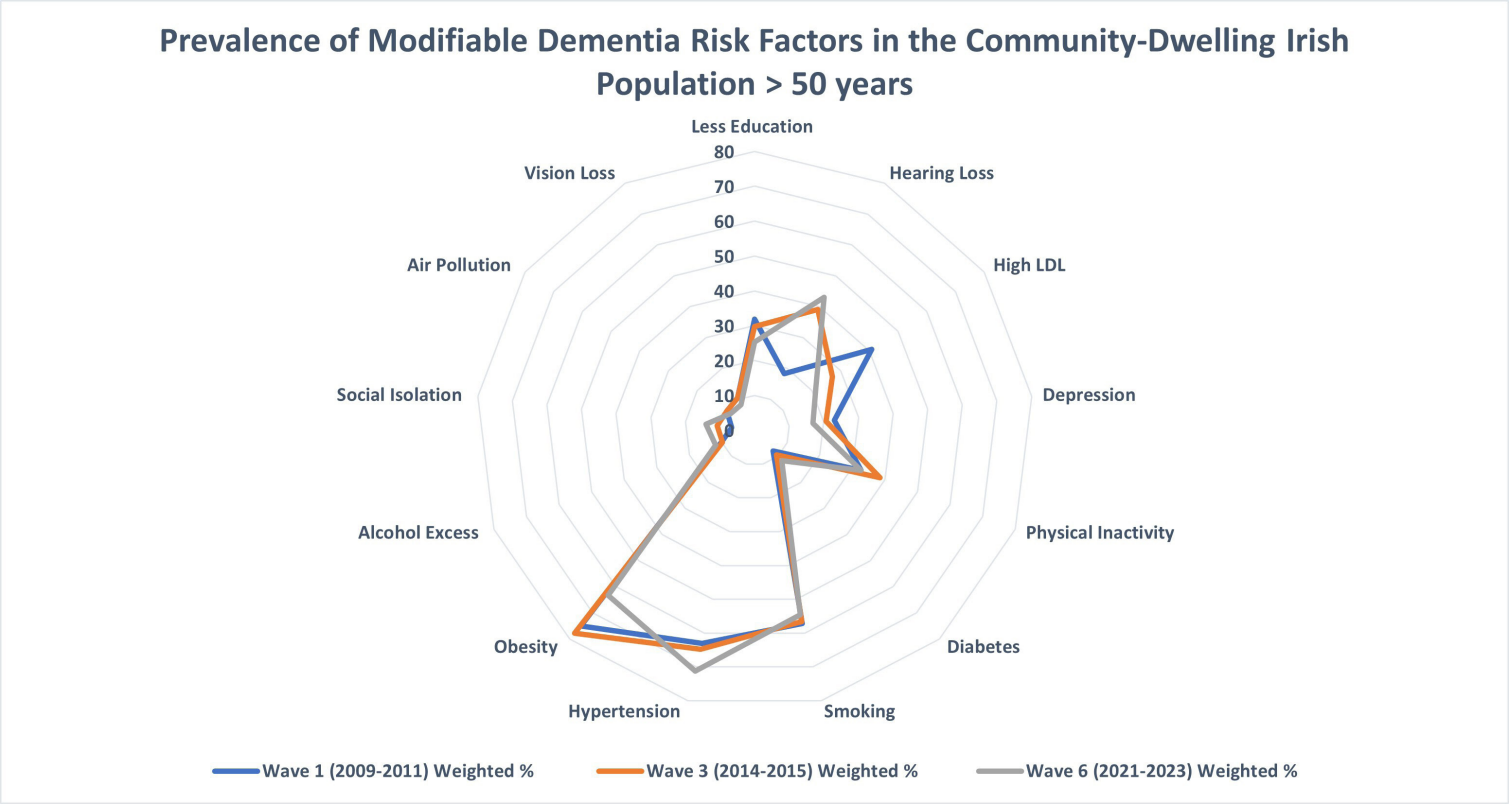
Prevalence of modifiable dementia risk factors in community-dwelling adults over 50 years in Ireland. This radar chart depicts prevalence of individual modifiable dementia risk factors in community-dwelling adults over 50 years in Ireland at three time points: wave 1 (2009–2011), wave 3 (2014–2015) and wave 6 (2022–2023). The blue line represents the data from wave 1, the orange line represents the data from wave 3 and the grey line represents data from wave 6. Each axis represents a specific modifiable risk factor for dementia and data points are presented as weighted prevalence at each wave, allowing the visualisation of the change in prevalence of individual risk factors over 10 years of follow-up. LDL, low-density lipoprotein.

Risk factors were also examined on an individual basis with the results displayed in table 4 and figure 2. Regarding environmental and social factors, 1 in 12 older people were exposed to air pollution at wave 6, which did not change significantly across waves, while the prevalence of social isolation rose from 6.6% at wave 1, to 10.9% at wave 3 and 14% at wave 6. Results show a high level of cardiovascular risk factors with elevated rates of HTN, obesity, smoking and high LDL cholesterol. A high level of physical inactivity was also evident.

**Table 4:** Prevalence of modifiable dementia risk factors

Risk factor	Wave 1 weighted % and CI	Population count	Wave 3 weighted % and CI	Population count	Wave 6 weighted % and CI	Population count
Less education	31.8 (30.4 to 33.4)	403 848	29.7 (28.1 to 31.3)	346 068	25.3 (23.5 to 27.2)	260 643
Hearing loss	18.3 (17.3 to 19.3)	231 956	39.1 (37.8 to 40.5)	456 217	43.0 (41.2 to 44.8)	442 889
High LDL	40.9 (39.4 to 42.3)	518 070	27.1 (25.6 to 28.7)	322 845	21.4 (19.1 to 23.9)	219 305
Depression	23.0 (21.9 to 24.1)	291 082	20.6 (19.5 to 21.8)	240 294	16.9 (15.5 to 18.4)	174 002
Physical inactivity	32.7 (31.3 to 34.2)	414 796	38.5 (36.8 to 40.2)	448 770	32.7 (30.8 to 34.6)	336 987
Diabetes	7.9 (7.4 to 8.6)	100 678	9.5 (8.7 to 10.3)	110 510	11.7 (10.6 to 12.9)	120 582
Smoking	57.1 (55.8 to 58.4)	724 454	56.6 (55.3 to 58.3)	662 322	54.6 (52.8 to 56.4)	562 806
Hypertension	63.0 (61.6 to 64.4)	798 944	64.7 (63.1 to 66.3)	769 871	71.2 (68.5 to 73.8)	729 306
Obesity	75.1 (73.9 to 76.3)	952 561	77.8 (76.4 to 79.2)	926 237	63.3 (60.2 to 66.3)	648 680
Alcohol excess	10.3 (9.6 to 11.0)	130 243	9.8 (9.0 to 10.7)	114 392	11.9 (10.7 to 13.1)	122 219
Social isolation	6.6 (6.0 to 7.3)	83 927	10.9 (10.0 to 12.0)	127 357	14.0 (12.6 to 15.5)	144 326
Air pollution	9.7 (8.7 to 10.9)	120 210	9.7 (8.6 to 10.9)	100 481	8.3 (7.2 to 9.7)	86 117
Vision loss	10.4 (9.6 to 11.2)	131 393	10.4 (9.5 to 11.4)	121 123	8.3 (7.3 to 9.5)	85 780

LDL, low-density lipoprotein.

**Figure 3:**
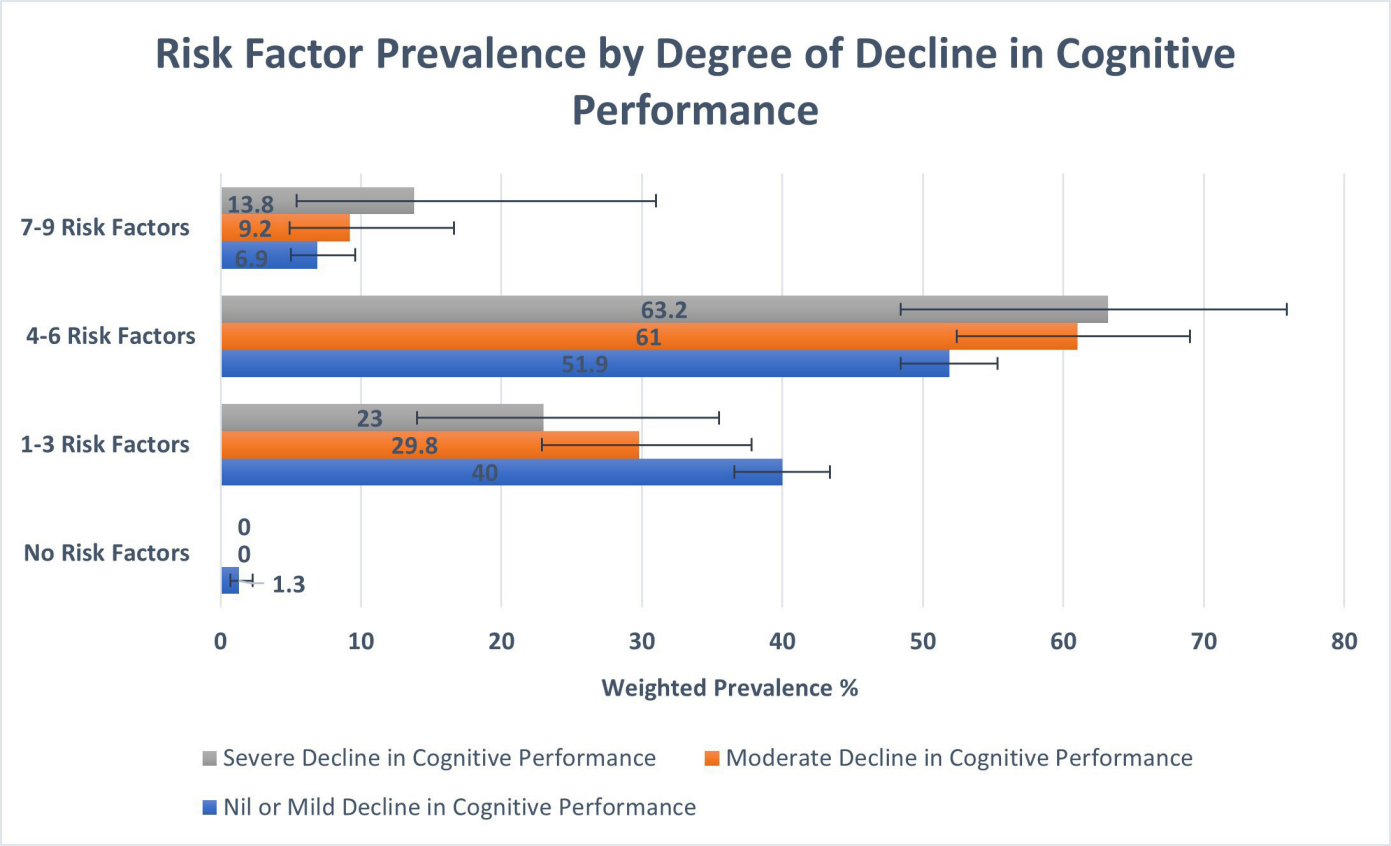
Risk factor prevalence by degree of decline in cognitive performance. This bar chart depicts the prevalence of modifiable dementia risk factors at baseline in wave 1, compared by the degree of cognitive decline over the follow-up period. The blue bars represent the data for those with no or mild cognitive decline, the orange bars represent those with moderate cognitive decline and the grey bars represent those with severe cognitive decline. The number of risk factors is displayed on the y-axis and weighted prevalence for each group, expressed as a percentage, is displayed on the x-axis. Error bars represent the 95% CIs for each group. As seen on the y-axis, risk factor groups are as follows: no risk factors, 1–3 risk factors, 4–6 risk factors and 7–9 risk factors.

On weighted analysis of decline in cognitive performance from baseline in this study, 224 938 people or 24.9% of the population experienced moderate or severe decline during the follow-up period. As demonstrated in figure 3, those with severe decline in cognitive performance had higher numbers of modifiable risk factors at wave 1 compared with those with nil/mild decline and those with moderate decline. Of those with moderate to severe decline in cognitive performance, only 39.3% self-reported an awareness that their memory was ‘a bit worse’ or ‘much worse’ compared with the last time they were interviewed. As per the Lancet Commission, up to 45% of dementia cases could be prevented by eliminating modifiable risk factors. Applying this estimate to those with a degree of decline in cognitive performance in this study would indicate that a possible 68 742 cases of moderate and 32 480 cases of severe decline in cognitive performance could have been prevented over the 11 years of follow-up in this study. In 2019, Pierse *et al* published estimates for dementia prevalence in Ireland with numbers ranging from 39 272 to 55 266 depending on the source used.^18^ Applying the same principle to these estimates would indicate that between approximately 17 500 - 25 000 of these dementia cases were preventable.

As demonstrated in table 5, rates of intervention were examined for risk factors with direct and measurable treatment options. Of those with hearing loss, 18.7% in wave 1, 14.0% in wave 3 and 14.5% in wave 6 reported using hearing aids all the time. 94.2%, 61.9% and 67.4% of those with vision loss reported wearing glasses or contact lenses most of the time in waves 1, 3 and 6, respectively. In terms of treatment for depression at wave 3, 48.1% were taking antidepressant medication, but only 3.0% of those asked had received any psychological intervention. Antidepressant use in the setting of depression rose to 74.7% in wave 6.

**Table 5:** Risk factors with direct and quantifiable interventions

Risk factor and intervention	Wave 1 weighted % and CI	Wave 3 weighted % and CI	Wave 6 weighted % and CI
Hearing loss			
Hearing aids (using all the time)	18.7 (16.0 to 21.8)	14.0 (12.6 to 15.7)	14.5 (12.7 to 16.6)
Hearing aids (occasional use)	12.5 (10.5 to 14.8)	8.5 (7.3 to 9.9)	13.9 (12.1 to 15.9)
Vision loss			
Glasses/lenses	94.2 (93.2 to 95.0)	61.9 (57.1 to 66.4)	67.4 (60.3 to 73.9)
Cataracts	13.3 (12.0 to 14.7)	28.2 (24.4 to 32.4)	34.7 (28.9 to 41.0)
Glaucoma	2.9 (2.4 to 3.6)	8.5 (6.4 to 11.2)	11.8 (8.3 to 16.5)
Age-related macular degeneration	2.5 (1.9 to 3.2)	12.5 (9.6 to 16.2)	15.9 (11.8 to 21.2)
Diabetes			
Diabetic medications	72 (67.1 to 76.6)	78.8 (74.9 to 82.3)	83.6 (79.3 to 87.1)
Depression			
Antidepressant medication	26.2 (23.8 to 28.8)	48.1 (44.9 to 51.3)	74.7 (70.5 to 78.6)
Psychological intervention	6.2 (4.9 to 7.8)	3.0 (2.1 to 4.2)	3.1 (2.0 to 4.8)
Hypertension			
Antihypertensive medication	60.2 (58.4 to 62.0)	70.0 (67.7 to 71.5)	71.6 (68.2 to 74.8)
High LDL cholesterol			
Lipid-lowering therapy	16.7 (15.0 to 18.6)	14.1 (12.1 to 16.3)	15.2 (10.7 to 21.1)

LDL, low-density lipoprotein.

Regarding cardiovascular risk factors at wave 3, 78.8% of those with a self-reported diagnosis of diabetes were on a diabetes medication, 70% of those with hypertension identified in the HA were receiving an antihypertensive medication and 14.1% of those with high LDL cholesterol were on a lipid lowering treatment. Control of cardiovascular risk factors was also examined. Of those on treatment for hypertension, 53.2% were controlled to a target BP of less than 140/90. Regarding those on lipid-lowering treatment, 77.1% had their LDL level reduced below 3 mmol/L while 64.8% of those on medication for diabetes achieved a target haemoglobin A1c (HbA1c) of 53 mmol/mol or less. Concerningly, only 37.7% of those with an HbA1c>48 mmol/mol self-reported a diagnosis of diabetes, perhaps indicating a high level of undiagnosed diabetes.

## Discussion

This study demonstrates a high prevalence of modifiable risk factors for dementia, as described by the 2024 Lancet Commission on dementia, in community-dwelling adults over 50 years old in Ireland. 70.6% of the population at wave 1, 61.1% at wave 3 and 54.2% at wave 6 had four or more risk factors for dementia; this amounted to over half a million people at each wave on weighted analysis as displayed in table 2. Previous research on modifiable dementia risk factors in Ireland has found that the distribution of risk factor exposure as well as the barriers to risk factor management vary across gender, age and education groups and also that public awareness of modifiable risk factors for dementia differs according to risk factor, gender and level of educational attainment.^20 21^

Of particular concern are the persistent and increasingly high levels of cardiovascular risk factors that this study identifies across each wave. Hypertension prevalence ranged from 63.0% to 71.2% and increased from wave to wave. LDL cholesterol was elevated >3 mmol/L in 40.9%, 27.1% and 21.4% of the population at waves 1, 3 and 6, respectively. In terms of obesity, 75.1% of the population at wave 1 and 77.8% at wave 3 were obese as defined by their measured BMI and/or waist-hip ratio. Obesity at wave 6 was measured by BMI and waist circumference and levels reduced to 63.3%. The prevalence of diabetes increased across waves, affecting 7.9% in wave 1 compared with 9.5% in wave 3 and 11.7% in wave 6.

It is well documented that each of these conditions contributes significantly to the risk of developing dementia in later life.^5 22^ The prevalence of hypertension in Ireland has been found to be similar in previous studies. The total prevalence of hypertension in adults over 45 years old was 62% when estimated by Barron *et al* using data from SLÁN in 2007^23^ and 63.7% by Murphy *et al*.^16^ In terms of the prevalence of high LDL cholesterol, a study conducted in Ireland in 2017 found that 60% of the population studied, with a mean age of 51 years, had significant lipid abnormalities with 58% specifically having an LDL cholesterol >3 mmol/L.^24^ The prevalence of obesity in Ireland was found to be 23% in 2019, this lower prevalence, when compared with our study, may be explained by the fact that the 2019 study only included BMI-classified obesity and was based on the entire adult population as opposed to adults aged 50 years and older in TILDA.^25^ The prevalence of diabetes in this age group has been found to be similar in previous studies.^26 27^

Regarding the risk factors related to sensory loss, hearing loss was identified in 18.3% of the population at wave 1, increasing to 39.1% at wave 3 and 43.0% at wave 6 with the addition of a further question in the CAPI specifically asking the participant if they experienced any hearing loss. The prevalence of hearing impairment based on previous studies using data from Census 2011 and 2016, the National Disability Survey 2006, and the Irish Health Survey 2015 as well as previous studies within TILDA has been in keeping with the findings in this study.^28 29^ The prevalence of visual loss was consistent across the waves. According to the 2022 Census, a total of 296 601 people experienced blindness or vision impairment to any extent, increasing significantly with age.^30^

In terms of mental health and lifestyle factors, depression was found to be a risk factor in 23% of the population at wave 1, 20.6% at wave 3 and 16.9% at wave 6. The prevalence of major depressive disorder was 11.5% in a study published in 2022, based on a cohort of adults over 18 years old.^31^ Social isolation increased across the waves from 6.6% at wave 1 to 14.0% at wave 6. The impact of the COVID-19 pandemic on levels of social isolation and loneliness has been well-documented.^32^ The prevalence of smoking and excess alcohol consumption was consistent across the waves, with smoking rates ranging from 54.6% to 57.1% and excess alcohol intake ranging from 9.8% to 11.9%. 2022 Census data found that 18.9% of the population were ex-smokers, 8.7% were current daily smokers and 4.4% were occasional smokers.^30^ A Health Research Board report in 2021, stated that one-third of adults over 65 who consume alcohol were hazardous drinkers, with 40% of men over 65 engaging in monthly binge drinking.^33^

Air pollution and less educational attainment were also identified by the Lancet Commission on dementia as modifiable risk factors for dementia. The prevalence of exposure to air pollution was found to be between 8.3% and 9.7% across the three waves; however, this is likely to be significantly underestimated as it was only possible to account for one source of air pollution in this study. A report published by the Environmental Protection Agency in 2022 on air quality in Ireland found that most of Ireland did not meet the WHO guideline for fine particulate matter levels of 5 µg/m^3^ and highlighted with modelling that elevated concentrations of nitric oxide existed along the major urban road network in Ireland’s larger cities. Levels of educational attainment improved across the three waves, falling from 31.8% in wave 1 to 25.3% in wave 6. This pattern was also demonstrated in the 2022 census which showed that educational attainment has improved steadily between 1991 and 2022.^34^

Regarding appropriate interventions for these modifiable risk factors, this study identifies the need for a renewed focus on the management of cardiovascular risk factors as well as appropriately addressing sensory loss. This is particularly important in terms of primary prevention. The low levels of hearing aid use in those that self-rated their hearing as poor or fair are likely due to a number of issues. Recent reports and research have highlighted several barriers to receiving a hearing aid, including cost, lack of insurance coverage, concerns regarding appearance and lack of emphasis on the issue from healthcare providers.^29^ From a cardiovascular point of view, there is room for significant improvement in the diagnosis, treatment and control of hypertension, high-LDL cholesterol and diabetes as demonstrated in this study and others.^16 35^

Decline in cognitive performance was assessed over a mean follow-up period of 10.93 (±0.37) years. 77% of those that developed a severe decline in cognitive performance by wave 6 had four or more modifiable risk factors present at wave 1. Figure 3 demonstrates that the increasing numbers of modifiable risk factors were found to be proportional to the degree of decline in cognitive performance. Adequately addressing the modifiable risk factors identified by the Lancet Commission has the potential to dramatically alter the future landscape of dementia care in Ireland. There is a need for greater attention on educating people on the concept of brain health through public health messaging, and the development of a clinical framework via brain health clinics and memory services to better deliver on the opportunity of dementia prevention as recommended by a recent European task force.^36^ By focussing on these risk factors at a national level and placing an emphasis on the importance of brain health at all stages of life, the incidence of dementia could be significantly reduced and its onset delayed.

The strengths of this study include the involvement of a large, population-representative cohort of community-dwelling older adults. Comprehensive data collection, standardised HAs and ongoing follow-up with extensive longitudinal data further enhance the robustness of this study. This study has some limitations; some of the variables, including established CVD and diabetes status, are based on participants’ self-report of a doctor-delivered diagnosis, which could be subject to recall bias. In terms of hypertension measurement, it is well documented that BP readings can be inappropriately high or low in healthcare settings, termed either ‘white coat hypertension’ or ‘masked hypertension’, this may cause overestimation or underestimation of hypertension prevalence.^37 38^ Additionally, exposure to air pollution was likely underestimated as only one source of indoor air pollution was examined in this study. In terms of the attrition rate between waves, this can be explained by a combination of mortality, participants withdrawing from the study and participants opting in or out of certain waves. Furthermore, cognitive decline often causes participants to withdraw from longitudinal studies, and this may lead to an underestimation of dementia risk.

In conclusion, this study identifies a high prevalence of individual and combined modifiable risk factors for dementia as described by the Lancet Commission on dementia. Furthermore, these results demonstrate significant room for improvement in terms of targeted interventions for the risk factors highlighted. Given that the Lancet Commission estimates that eliminating these risk factors could reduce cases of dementia by 45%, it is crucial that they are prioritised in national policies going forward. To drive meaningful change, it is essential that sufficient resources are allocated to tackle these risks effectively, creating a powerful opportunity to reshape the future of dementia care in Ireland.

## Data Availability

Data are available upon reasonable request.

## References

[1] Organisation WH (2017). Global action plan on the public health response to dementia 2017 - 2025.

[2] Organisation WH Dementia fact sheet 2023. https://www.who.int/news-room/fact-sheets/detail/dementia.

[3] Nichols E, Steinmetz JD, Vollset SE (2022). Estimation of the global prevalence of dementia in 2019 and forecasted prevalence in 2050: an analysis for the Global Burden of Disease Study 2019. Lancet Public Health.

[4] Wolters FJ, Chibnik LB, Waziry R (2020). Twenty-seven-year time trends in dementia incidence in Europe and the United States: The Alzheimer Cohorts Consortium. Neurology (ECronicon).

[5] Livingston G, Huntley J, Liu KY (2024). Dementia prevention, intervention, and care: 2024 report of the Lancet standing Commission. The Lancet.

[6] Whelan B (1979). Ransam - random sample design for Ireland. Econ Soc Res Inst.

[7] Cronin H, O’Regan C, Finucane C (2013). Health and aging: development of the Irish Longitudinal Study on Ageing health assessment. J Am Geriatr Soc.

[8] Kenny Gibson W, Cronin H, Kenny RA (2014). Validation of the self-reported hearing questions in the Irish Longitudinal Study on Ageing against the Whispered Voice Test. BMC Res Notes.

[9] Ewing JA (1984). Detecting alcoholism. The CAGE questionnaire. JAMA.

[10] Craig CL, Marshall AL, Sjöström M (2003). International physical activity questionnaire: 12-country reliability and validity. Med Sci Sports Exerc.

[11] Berkman LF, Syme SL (1979). Social networks, host resistance, and mortality: a nine-year follow-up study of Alameda County residents. Am J Epidemiol.

[12] Sheehan TJ, Fifield J, Reisine S (1995). The measurement structure of the Center for Epidemiologic Studies Depression Scale. J Pers Assess.

[13] Briggs R, Carey D, O’Halloran AM (2018). Validation of the 8-item Centre for Epidemiological Studies Depression Scale in a cohort of community-dwelling older people: data from The Irish Longitudinal Study on Ageing (TILDA). Eur Geriatr Med.

[14] O’Halloran AM, Kenny RA, King-Kallimanis BL (2014). The latent factors of depression from the short forms of the CES-D are consistent, reliable and valid in community-living older adults. Eur Geriatr Med.

[15] Ross R, Neeland IJ, Yamashita S (2020). Waist circumference as a vital sign in clinical practice: a Consensus Statement from the IAS and ICCR Working Group on Visceral Obesity. Nat Rev Endocrinol.

[16] Murphy CM, Kearney PM, Shelley EB (2016). Hypertension prevalence, awareness, treatment and control in the over 50s in Ireland: evidence from The Irish Longitudinal Study on Ageing. J Public Health (Oxf).

[17] Kenny RA, Coen RF, Frewen J (2013). Normative values of cognitive and physical function in older adults: findings from the Irish Longitudinal Study on Ageing. J Am Geriatr Soc.

[18] Pierse T, O’ Shea E, Carney P (2019). Estimates of the prevalence, incidence and severity of dementia in Ireland. Ir j psychol Med.

[19] Whelan BJ, Savva GM (2013). Design and methodology of the Irish Longitudinal Study on Ageing. J Am Geriatr Soc.

[20] Dukelow T, Lawrence EG, Jacobson L (2022). Modifiable risk factors for dementia, and awareness of brain health behaviors: Results from the Five Lives Brain Health Ireland Survey (FLBHIS). Front Psychol.

[21] Dukelow T, Vassilev P, Lawrence EG (2023). Barriers to brain health behaviours: results from the Five Lives Brain Health Ireland Survey. Front Psychol.

[22] Rolandi E, Zaccaria D, Vaccaro R (2020). Estimating the potential for dementia prevention through modifiable risk factors elimination in the real-world setting: a population-based study. *Alz Res Therapy*.

[23] Barron S, Balanda K, Hughes J (2014). National and subnational hypertension prevalence estimates for the Republic of Ireland: better outcome and risk factor data are needed to produce better prevalence estimates. BMC Public Health.

[24] Agar R, Markham C, Prendergast M (2019). A snapshot of lipid levels in the Republic of Ireland in 2017. Ir J Med Sci.

[25] Murrin C, Courtney AHJ (2022). ASOI adult obesity clinical practice guideline adaptation: association for the study of obesity on the island of Ireland.

[26] Balanda KP, Buckley CM, Barron SJ (2013). Prevalence of diabetes in the Republic of Ireland: results from the National Health Survey (SLAN) 2007. PLoS One.

[27] Leahy S, O’ Halloran AM, O’ Leary N (2015). Prevalence and correlates of diagnosed and undiagnosed type 2 diabetes mellitus and pre-diabetes in older adults: Findings from the Irish Longitudinal Study on Ageing (TILDA). Diabetes Res Clin Pract.

[28] Creedon Y (2017). The prevalence of self-reported hearing loss in Munster.

[29] Christine McGarrigle OD (2023). The Irish longitudinal study on ageing.

[30] Office CS (2023). Census 2022 profile 4 - disability, health and carers. https://www.cso.ie/en/releasesandpublications/ep/p-cpp4/census2022profile4-disabilityhealthandcarers/typeofdisability/.

[31] Hyland P, Vallières F, Shevlin M (2022). State of Ireland’s mental health: findings from a nationally representative survey. Epidemiol Psychiatr Sci.

[32] Mark Ward CM, Hever A, O’Mahoney P (2020). Loneliness and social isolation in the COVID-19 pandemic among the over 70s: data from the Irish longitudinal study on ageing (TILDA) and ALONE. Online TILDA, ALONE.

[33] O’Dwyer CMD, Doyle A, Galvin B (2021). Alcohol consumption, alcohol-related harm and alcohol policy in Ireland. HRB overview series 11.

[34] Office CS (2023). Census of population 2022 profile 8 - the Irish language and education. https://www.cso.ie/en/releasesandpublications/ep/p-cpp8/censusofpopulation2022profile8-theirishlanguageandeducation/levelofeducation/.

[35] Murphy C, Shelley E, O’Halloran AM (2017). Failure to control hypercholesterolaemia in the Irish adult population: cross-sectional analysis of the baseline wave of The Irish Longitudinal Study on Ageing (TILDA). Ir J Med Sci.

[36] Frisoni GB, Altomare D, Ribaldi F (2023). Dementia prevention in memory clinics: recommendations from the European task force for brain health services. *Lancet Reg Health Eur*.

[37] Franklin SS, Thijs L, Hansen TW (2013). White-coat hypertension: new insights from recent studies. Hypertension.

[38] Thakkar HV, Pope A, Anpalahan M (2020). Masked Hypertension: A Systematic Review. Heart Lung Circ.

