# Supplementary material for: How prevalent are modifiable dementia risk factors in Ireland? A 12-year observational study in community-dwelling older adults
Source: BMJ Open. 2025 Nov 11;15(11):e106032. doi: 10.1136/bmjopen-2025-106032 (PMC12606470; doi:10.1136/bmjopen-2025-106032)
Supplement: Supplementary file 1 [file bmjopen-2025-106032.reviewer_comments.pdf]

PEER REVIEW HISTORY

BMJ Open publishes all reviews undertaken for accepted manuscripts. Reviewers are asked to complete a checklist review form and are provided with free text boxes to elaborate on their assessment. These free text comments are reproduced below.

ARTICLE DETAILS

Title (Provisional)

How prevalent are modifiable dementia risk factors in Ireland? A 12-year observational study in community-dwelling older adults.

Authors

McGarvey, Caoimhe; Kenny, RoseAnne; Kennelly, Sean; Sexton, Donal; Briggs, Robert

---

VERSION 1 - REVIEW

**Reviewer**	**1**
**Name**	**Bray, Jennifer**
**Affiliation**	**University of Worcester**
**Date**	**17-Jul-2025**
**COI**	**None**

Page 2, abstract, objectives – I'm not sure I agree with some of the language used as it feels quite definite and clear cut. For example, saying 'dementia is preventable' gives a false impression as it implies that doing/not doing something will mean you don't get dementia, when actually it's about reducing the chance of developing dementia and addressing risk factors. The Lancet report you refer to uses language such as 'around 45% of cases of dementia are potentially preventable by addressing 14 modifiable risk factors' which is more nuanced and less definitive. Also, is addressing risk factors the same as 'eliminating' them? I'm not saying it's wrong, but it just makes your study seem black and white and like it's an all or nothing approach (with the potential to blame people for developing dementia), when I think there is a lot more going on.

Page 5, lines 37-39 – I find the sentence 'Participants with a doctor's diagnosis…consent to participate' concerning as it implies that once someone is diagnosed with dementia or cognitive impairment they lose capacity to consent. Not only does this blanket statement ignore individual situations, but it suggests that people with dementia (regardless of stage) cannot take part in research without someone else giving consent on their behalf. I'm sure there are many researchers and participants who would disagree with this. I have no

objection to people being excluded because of their diagnosis as it might affect the findings, it's the reason you gave on not being able to consent that I object to.

Page 5, risk factor measurement and Table 1 – your text implies that Table 1 indicates that risk factors were recorded in the CAPI, but Table 1 makes no mention of CAPI. You also mention lots of other measures and questionnaires for individual risk factors, but previously you only mentioned the CAPI, SCQ and HA, so it's not clear how these relate to each other. For example, was the IPAQ part of the CAPI, SCQ or HA? You mention it for some elements, such as CES-D being within the CAPI, so why not for all? Also, it would be useful if the order that the risk factors are discussed in the text matched the order they are presented in Table 1 to make it easier for the reader to cross check everything and get clarity. Or maybe group them according to whether they were measured in the CAPI, SCQ or HA?

If the use of hearing aids was part of the hearing loss measure/reporting, why was the use of glasses/contact lenses not part of the visual loss measure/reporting? I can see that later in the covariates section you say that 'Participants reported in the CAPI if they wore hearing aids all the time or occasionally and if they usually wore glasses or contact lenses', so I don't know if it's just how you've described it in the text (although Table 1 makes no mention of glasses/contacts), but it seems odd that different approaches were used.

Page 7, final paragraph before Table 1 – this is an odd paragraph as it covers three separate topics. Firstly, hypertension and blood pressure, which feels more relevant to be part of the previous paragraph where you have started talking about blood pressure. Secondly, traumatic brain injury, although I realise that as one very short sentence it needs to fit with something else rather than being on its own. Thirdly, the categories for number of risk factors, which feels like it should be separate as it's not about how the individual risk factors were measured like the rest of the section, but more about how the results were processed.

Page 11, results – I'm not sure how dependency levels and ADL impairment fit with anything as you haven't discussed capturing relevant information for this element, but introduce it as something you're measured in the results. Where has this come from? If it's a terminology issue and it's related to another measure or risk factor, this needs to be made clear.

Figure 1 – I don't have strong feelings either way, but it feels like this doesn't really add anything as all the information is available in Table 3. I know it presents key information in a more visual way, but is it needed?

Figure 2 – again, it's nice to be able to see the information in a different way, but does it add anything? If anything, it makes me question some of the text in the paragraph before Table 4 where you talk about the results. You say there are 'high levels of hearing loss and moderate levels of visual loss' but in Figure 4 hearing loss is mid-range at best, and visual loss barely registers. Do you have definitions for 'high' and 'moderate' that would help, as these are quite subjective terms that different readers would interpret differently. Similarly, you say 'persistent prevalence of air pollution and increasing prevalence of social isolation' and while technically true (air pollution is consistent at around 8-10% and social isolation does increase

over time), they are barely visible in Figure 2, making it feel like you're drawing attention to something quite minor. I know you can't really mention everything, but it seems odd to focus on those factors rather than physical activity which is a lot higher than both air pollution and social isolation combined. You also talk about 'a high level of cardiovascular risk factors with elevated rates of hypertension, obesity, and high LDL cholesterol', and again this is true, but why no mention of smoking which is a lot higher than the high LDL? If you're not counting smoking as a cardiovascular risk but a lifestyle factor (although the internet suggests smoking is a major cause of cardiovascular disease), it still feels worthy of a mention.

Figure 3 – the text on page 14 suggests that this relates to wave one, but there is nothing in the Figure 3 title or information to confirm this, so if the reader missed the text it would be easy to be confused about what Figure 3 is showing, or misinterpret it.

Page 16, discussion – when giving the diabetes figures you say 11.9% for wave six, but Table 4 says 11.7%. Which is correct?

Page 17, discussion – you say 'The prevalence of smoking and excess alcohol consumption was consistent across the waves' but without extra context this feels a bit misleading as it could be taken to imply that they were similar when actually smoking is around 55-57% and alcohol excess is down at 10-12%. You've given a lot of the other results, so it might be worth doing the same here to enable the reader to have the full picture without having to go back to Table 4.

**Reviewer**	**2**
**Name**	**Leist, Anja**
**Affiliation**	**University of Luxembourg, Department of Social Sciences**
**Date**	**23-Jul-2025**
**COI**	**None**

This study assesses the prevalence of modifiable risk factors in the TILDA panel survey in Ireland, and estimates the contribution of modifiable risk factors to severe cognitive decline. The study is situated in the Lancet Commission's framework of modifiable risk factors for dementia, and uses mostly appropriate caution in estimating the number of cases of preventable severe cognitive decline. It is an important contribution to understand risk factor burden in the Irish population aged 50 and older. I have a few comments.

METHODS

p. 4 line 17 ff. "Each member of the population in Ireland aged 50 years and older had an equal probability of being invited to participate." Please add information whether only non-institutionalised adults were sampled, similar to the SHARE survey?

The information whether a variable is recorded in the CAPI is irrelevant in the data analysis, I suggest to remove these notions.

The method section lays out the assessment of risk factors. Many of these are assessed differently than how the Lancet Commission has operationalised the modifiable risk factors, e.g. alcohol consumption in TILDA is assessed via the CAGE questionnaire which identifies harmful drinking, whereas the Lancet Commission speaks about alcohol consumption in number of units, specifically more than 21 UK units per week (Livingston et al., 2020, 2024). CAGE scores and number of units of alcohol consumed may correlate but still address the issue of alcohol consumption differently.

The variable air pollution is self-reported as "risk factor in those that self-reported using an open fire or a combination of an open fire and portable heater to heat their home during winter in the SCQ.", and the reason why the participant residence could not linked to air pollution scores should be mentioned in the manuscript.

I suggest to include a comparison between assessment of risk factors in the evidence summarised by the Lancet Commission and the assessment in the TILDA study across all investigated risk factors.

Regarding the operationalisation of cognitive decline, I would recommend to add information whether only two assessments of cognition at baseline and at last follow-up were used. Was the baseline wave the first wave of assessment for each participant, e.g. were participants in wave 3 all newly entering the study, and if not, was there an adjustment for the practice effects at the second assessment? How were participants treated who participated in all observed waves 1, 3, and 6, or did participants' assessments count only towards risk factor prevalence at wave 1 or wave 3?

DISCUSSION

The prevalence of cognitive decline is an important finding, but the observational finding that the presence of modifiable risk factors is associated with cognitive decline needs to be presented more carefully; so far there is not much evidence that by addressing already present risk factors risk of dementia or risk of cognitive decline can be modified. The Lancet Commission to the most part relies on epidemiological evidence where presence of a risk factor is associated with increased dementia risk, i.e. primary prevention can be considered effective, while few interventional studies exist that have found secondary prevention to be effective (there is evidence on hypertension from the SPRINT-MIND trial but e.g. little evidence for the use of hearing aids to address hearing loss in the ACHIEVE trial). I suggest to frame the estimated gains from addressing risk factors specifically in terms of primary prevention in future cohorts, or keeping the current cohorts as healthy as possible by trying for them to accumulate more risk factors.

MINOR POINTS

The abbreviation HTN should be introduced/replaced by the full term in the highlights section.

VERSION 1 - AUTHOR RESPONSE

Reviewer: 1
Ms. Jennifer Bray, University of Worcester
Comments to the Author:
Page 2, abstract, objectives – I'm not sure I agree with some of the language used as it feels quite definite and clear cut. For example, saying 'dementia is preventable' gives a false impression as it implies that doing/not doing something will mean you don't get dementia, when actually it's about reducing the chance of developing dementia and addressing risk factors. The Lancet report you refer to uses language such as 'around 45% of cases of dementia are potentially preventable by addressing 14 modifiable risk factors' which is more nuanced and less definitive. Also, is addressing risk factors the same as 'eliminating' them? I'm not saying it's wrong, but it just makes your study seem black and white and like it's an all or nothing approach (with the potential to blame people for developing dementia), when I think there is a lot more going on.

Thank you for highlighting this, we have adjusted the language used as advised: 'Dementia is potentially preventable and deferrable yet remains a major cause of disability, dependency and mortality worldwide. The 2024 Lancet Commission on dementia identified 14 modifiable dementia risk factors and estimated that addressing these could reduce dementia cases by up to 45%.'

Page 5, lines 37-39 – I find the sentence 'Participants with a doctor's diagnosis...consent to participate' concerning as it implies that once someone is diagnosed with dementia or cognitive impairment they lose capacity to consent. Not only does this blanket statement ignore individual situations, but it suggests that people with dementia (regardless of stage) cannot take part in research without someone else giving consent on their behalf. I'm sure there are many researchers and participants who would disagree with this. I have no objection to people being excluded because of their diagnosis as it might affect the findings, it's the reason you gave on not being able to consent that I object to.

Apologies, this sentence has now been adjusted: 'Participants with a doctor's diagnosis of dementia or cognitive impairment were excluded at wave one.'

Page 5, risk factor measurement and Table 1 – your text implies that Table 1 indicates that risk factors were recorded in the CAPI, but Table 1 makes no mention of CAPI. You also mention lots of other measures and questionnaires for individual risk factors, but previously you only mentioned the CAPI, SCQ and HA, so it's not clear how these relate to each other. For example, was the IPAQ part of the CAPI, SCQ or HA? You mention it for some elements, such as CES-D being within the CAPI, so why not for all? Also, it would be useful if the order that the risk factors are discussed in the text matched the order they are presented in Table 1 to make it easier for the reader to cross check everything and get clarity. Or maybe group them according to whether they were measured in the CAPI, SCQ or HA?

Sorry for the confusion, yes these questionnaires all formed part of the CAPI. I have now included the data source (CAPI/SCQ/HA) in Table 1, re-ordered the table in keeping with the text and grouped the risk factors according to whether they were measured in CAPI/SCQ/HA to provide more clarity.

If the use of hearing aids was part of the hearing loss measure/reporting, why was the use of glasses/contact lenses not part of the visual loss measure/reporting? I can see that later in the covariates section you say that 'Participants reported in the CAPI if they wore hearing aids all the time or occasionally and if they usually wore glasses or contact lenses', so I don't know if it's just how you've described it in the text (although Table 1 makes no mention of glasses/contacts), but it seems odd that different approaches were used.

Thank you for highlighting this, we did include the use of glasses and contact lenses as a measure of visual loss. The text has now been corrected to reflect this: **'Self-rated vision and usual use of glasses or contact lenses were recorded at the CAPI and visual loss was defined as a rating of poor or fair.'**

Page 7, final paragraph before Table 1 – this is an odd paragraph as it covers three separate topics. Firstly, hypertension and blood pressure, which feels more relevant to be part of the previous paragraph where you have started talking about blood pressure. Secondly, traumatic brain injury, although I realise that as one very short sentence it needs to fit with something else rather than being on its own. Thirdly, the categories for number of risk factors, which feels like it should be separate as it's not about how the individual risk factors were measured like the rest of the section, but more about how the results were processed.

Thank you, I have combined all blood pressure information into one paragraph and removed the risk factor categories piece to the analysis section within methods.

Page 11, results – I'm not sure how dependency levels and ADL impairment fit with anything as you haven't discussed capturing relevant information for this element but introduce it as something you're measured in the results. Where has this come from? If it's a terminology issue and it's related to another measure or risk factor, this needs to be made clear.

Apologies, this was an oversight. I have now discussed capturing ADL impairment in the covariate section of methods.

Figure 1 – I don't have strong feelings either way, but it feels like this doesn't really add anything as all the information is available in Table 3. I know it presents key information in a more visual way, but is it needed?

We thought it was helpful to have a visual representation of the change in risk factor prevalence over time but can certainly remove if required.

Figure 2 – again, it's nice to be able to see the information in a different way, but does it add anything? If anything, it makes me question some of the text in the paragraph before Table 4

where you talk about the results. You say there are 'high levels of hearing loss and moderate levels of visual loss' but in Figure 4 hearing loss is mid-range at best, and visual loss barely registers. Do you have definitions for 'high' and 'moderate' that would help, as these are quite subjective terms that different readers would interpret differently. Similarly, you say 'persistent prevalence of air pollution and increasing prevalence of social isolation' and while technically true (air pollution is consistent at around 8-10% and social isolation does increase over time), they are barely visible in Figure 2, making it feel like you're drawing attention to something quite minor. I know you can't really mention everything, but it seems odd to focus on those factors rather than physical activity which is a lot higher than both air pollution and social isolation combined. You also talk about 'a high level of cardiovascular risk factors with elevated rates of hypertension, obesity, and high LDL cholesterol', and again this is true, but why no mention of smoking which is a lot higher than the high LDL? If you're not counting smoking as a cardiovascular risk but a lifestyle factor (although the internet suggests smoking is a major cause of cardiovascular disease), it still feels worthy of a mention.

Thank you. We felt it was useful to have a visual representation of this data but can also remove if preferred. I have adjusted the text to avoid subjective terms, added smoking to the cardiovascular risk factors and highlighted the high rates of physical inactivity.

Figure 3 – the text on page 14 suggests that this relates to wave one, but there is nothing in the Figure 3 title or information to confirm this, so if the reader missed the text it would be easy to be confused about what Figure 3 is showing, or misinterpret it.

We have adjusted the Figure 3 legend to clarify that it refers to risk factors at baseline in wave one: 'This bar chart depicts prevalence of modifiable dementia risk factors at baseline in wave one compared by the degree cognitive decline over the follow period.'

Page 16, discussion – when giving the diabetes figures you say 11.9% for wave six, but Table 4 says 11.7%. Which is correct?

Apologies for the typo, 11.7% is correct and this has been corrected in the discussion section. Thank you.

Page 17, discussion – you say 'The prevalence of smoking and excess alcohol consumption was consistent across the waves' but without extra context this feels a bit misleading as it could be taken to imply that they were similar when actually smoking is around 55-57% and alcohol excess is down at 10-12%. You've given a lot of the other results, so it might be worth doing the same here to enable the reader to have the full picture without having to go back to Table 4.

This has now been clarified as follows: 'The prevalence of smoking and excess alcohol consumption was consistent across the waves, with smoking rates ranging from 54.6 - 57.1% and excess alcohol intake ranging from 9.8 - 11.9%.'

Reviewer: 2
Dr. Anja Leist, Universite du Luxembourg
Comments to the Author:
This study assesses the prevalence of modifiable risk factors in the TILDA panel survey in Ireland and estimates the contribution of modifiable risk factors to severe cognitive decline. The study is situated in the Lancet Commission's framework of modifiable risk factors for dementia and uses mostly appropriate caution in estimating the number of cases of preventable severe

cognitive decline. It is an important contribution to understand risk factor burden in the Irish population aged 50 and older. I have a few comments.

METHODS
p. 4 line 17 ff. "Each member of the population in Ireland aged 50 years and older had an equal probability of being invited to participate." Please add information whether only non-institutionalised adults were sampled, similar to the SHARE survey?
The information whether a variable is recorded in the CAPI is irrelevant in the data analysis, I suggest to remove these notions.

Thank you, I have reiterated here that only adults living in the community were included: '. Each member of the population in Ireland aged 50 years and older and living in the community had an equal probability of being invited to participate.'

The previous reviewer had requested clearer information regarding the CAPI/SCQ/HA and where data was collected so I have now added this information to Table 1. I can remove this from the text if that is preferred.

The method section lays out the assessment of risk factors. Many of these are assessed differently than how the Lancet Commission has operationalised the modifiable risk factors, e.g. alcohol consumption in TILDA is assessed via the CAGE questionnaire which identifies harmful drinking, whereas the Lancet Commission speaks about alcohol consumption in number of units, specifically more than 21 UK units per week (Livingston et al., 2020, 2024). CAGE scores and number of units of alcohol consumed may correlate but still address the issue of alcohol consumption differently.
The variable air pollution is self-reported as "risk factor in those that self-reported using an open fire or a combination of an open fire and portable heater to heat their home during winter in the SCQ.", and the reason why the participant residence could not linked to air pollution scores should be mentioned in the manuscript.
I suggest to include a comparison between assessment of risk factors in the evidence summarised by the Lancet Commission and the assessment in the TILDA study across all investigated risk factors.

Thank you. The participant's addresses are not routinely recorded as part of data collection. Geocodes are recorded but it was not possible to link them with an air pollution index.

The assessment & measurement of risk factors in the evidence summarised by the Lancet commission has now been included in Table 1 so that it can be compared easily to the measures used in TILDA.

Regarding the operationalisation of cognitive decline, I would recommend to add information whether only two assessments of cognition at baseline and at last follow-up were used. Was the baseline wave the first wave of assessment for each participant, e.g. were participants in wave 3 all newly entering the study, and if not, was there an adjustment for the practice effects at the second assessment? How were participants treated who participated in all observed waves 1, 3, and 6, or did participants' assessments count only towards risk factor prevalence at wave 1 or

wave 3?

Thank you, apologies for the lack of clarity here. Yes, only two assessments of cognition at baseline in wave 1 and at final follow up in wave 6 were used. The baseline wave was therefore the first wave of cognitive assessment for all participants. Furthermore, only baseline risk factor prevalence at wave 1 was included in this analysis. We have updated the text to reflect this: 'Risk factor prevalence for this analysis was measured at wave one and cognition was measured at wave one baseline and at wave six follow-up.'

DISCUSSION
The prevalence of cognitive decline is an important finding, but the observational finding that the presence of modifiable risk factors is associated with cognitive decline needs to be presented more carefully; so far there is not much evidence that by addressing already present risk factors risk of dementia or risk of cognitive decline can be modified. The Lancet Commission to the most part relies on epidemiological evidence where presence of a risk factor is associated with increased dementia risk, i.e. primary prevention can be considered effective, while few interventional studies exist that have found secondary prevention to be effective (there is evidence on hypertension from the SPRINT-MIND trial but e.g. little evidence for the use of hearing aids to address hearing loss in the ACHIEVE trial). I suggest to frame the estimated gains from addressing risk factors specifically in terms of primary prevention in future cohorts, or keeping the current cohorts as healthy as possible by trying for them to accumulate more risk factors.

Thank you, the text has been adjusted to focus on primary prevention: 'Regarding appropriate interventions for these modifiable risk factors, this study identifies the need for a renewed focus on the management of cardiovascular risk factors as well as appropriately addressing sensory loss. This is particularly important in terms of primary prevention' and 'Adequately addressing the modifiable risk factors identified by the Lancet Commission has the potential to dramatically alter the future landscape of dementia care in Ireland.'

MINOR POINTS
The abbreviation HTN should be introduced/replaced by the full term in the highlights section.
This has been corrected.

VERSION 2 - REVIEW

**Reviewer**	**1**
**Name**	**Bray, Jennifer**
**Affiliation**	**University of Worcester**
**Date**	**19-Aug-2025**

COI

Thank you for taking on board the comments from myself and the other reviewer. I only have one very minor comment still to raise.

Text between Tables 3&4 – I'm still not convinced of the need to talk about the 'persistent prevalence of air pollution and increasing prevalence of social isolation'. As in my previous comments I appreciate that what you say is technically true but neither goes above 14% so compared to other risk factors they are negligible. If you want to refer to these two risk factors, I think that you need to be clear that they are at very low prevalence levels as the text currently makes it feel like they are almost more important than other risk factors.

**Reviewer**	**2**
**Name**	**Leist, Anja**
**Affiliation**	**University of Luxembourg, Department of Social Sciences**
**Date**	**03-Sep-2025**
**COI**	

The reviewer comments have been sufficiently addressed, and the manuscript in my evaluation is ready for publication. One observation on the removal of the figures in R1: I don't have a strong opinion on these, but I think they can remain in the paper, particularly figure 2 which illustrates risk factor prevalence in the sample and thus possible entry points for primary prevention in that population. Apologies for the typo in my first review; the last sentence should of course read "... keeping the current cohorts as healthy as possible by supporting them to NOT accumulate more risk factors."

VERSION 2 - AUTHOR RESPONSE

Reviewer: 1
Ms. Jennifer Bray, University of Worcester
Comments to the Author:
Thank you for taking on board the comments from myself and the other reviewer. I only have one very minor comment still to raise.
Text between Tables 3&4 – I'm still not convinced of the need to talk about the 'persistent prevalence of air pollution and increasing prevalence of social isolation'. As in my previous comments I appreciate that what you say is technically true but neither goes above 14% so compared to other risk factors they are negligible. If you want to refer to these two risk factors, I think that you need to be clear that they are at very low prevalence levels as the text currently makes it feel like they are almost more important than other risk factors.

Thank you for this comment, to improve the clarity of this sentence we propose to change it to the following: 'Regarding environmental and social factors, 1 in 12 older people were exposed to air pollution at Wave 6, which did not change significantly across waves, while the prevalence of social isolation rose form 6.6% at Wave 1, to 10.9% at Wave 3 and 14% at Wave 6.'

Reviewer: 2
Prof. Anja Leist, University of Luxembourg
Comments to the Author:
The reviewer comments have been sufficiently addressed, and the manuscript in my evaluation is ready for publication. One observation on the removal of the figures in R1: I don't have a strong opinion on these, but I think they can remain in the paper, particularly figure 2 which illustrates risk factor prevalence in the sample and thus possible entry points for primary prevention in that population. Apologies for the typo in my first review; the last sentence should of course read "... keeping the current cohorts as healthy as possible by supporting them to NOT accumulate more risk factors."

Thank you for this comment.
